# Postural differences in the immediate effects of active exercise with compression therapy on lower limb lymphedema

**DOI:** 10.1007/s00520-020-05976-y

**Published:** 2021-04-29

**Authors:** Kiriko Abe, Tetsuya Tsuji, Asako Oka, Junichi Shoji, Michiyo Kamisako, Hiroka Hohri, Aiko Ishikawa, Meigen Liu

**Affiliations:** 1 Department of Rehabilitation Medicine, Keio University Graduate School of Medicine, 35 Shinanomachi, Shinjyuku-ku, Tokyo, 160-8582 Japan; 2 Department of Rehabilitation Medicine, Saiseikai Kanagawa Hospital, 6-6 Tomiyacho, Kanagawa-ku, Yokohama, Kanagawa-ken 221-0821 Japan; 3 Department of Rehabilitation Medicine, Keio University School of Medicine, 35 Shinanomachi, Shinjyuku-ku, Tokyo, 160-8582 Japan; 4 Department of Rehabilitation Medicine, Keio University Hospital, 35 Shinanomachi, Shinjyuku-ku, Tokyo, 160-8582 Japan

**Keywords:** Gynecological cancer, Lymphedema, Active exercise, Compression therapy, Rehabilitation

## Abstract

**Purpose:**

Although regarded as an important treatment for lymphedema, the therapeutic effects of active exercise with compression therapy (AECT) are supported by little evidence. The purpose of this study was to determine the relative benefits of AECT with different postures for patients with lower limb lymphedema (LLL).

**Methods:**

Eighteen women with LLL secondary to surgical treatment of gynecological cancer, completed (1) AECT in a seated position (seated AECT), (2) AECT in a supine position (supine AECT), and (3) compression-only therapy in a supine position (CT) in this randomized, controlled, crossover trial. AECT was performed on a bicycle ergometer while wearing elastic compression bandages. Each intervention was performed for 15 min, and the three conditions were separated by a 1-week washout period. Lower-limb volumes were evaluated using a Perometer^TM^ sensor (Pero-system, Wuppertal, Germany), and symptom severity was assessed before and after each intervention using a visual analog scale (pain, heaviness) and palpation (pitting, stiffness). The effects of the interventions were estimated using linear mixed-effect models.

**Results:**

The magnitude of limb volume decreases differed significantly among the interventions, with a greater decrease after supine AECT than after CT. Pre-intervention pitting severity and skin stiffness were significantly correlated with the magnitude of volume decrease after all interventions and after AECT in the supine position, respectively.

**Conclusions:**

Supine AECT using a bicycle ergometer has marked immediate effects to decrease the fluid volume of severe LLL.

**Clinical trial registration:**

UMIN clinical trial registry (UMIN-CTR; ID000020129) by CONSORT 2010, TRN R000023253, December 9, 2015

## Introduction

Lymphedema is the accumulation of high protein fluid in the interstitium due to the failure of lymphatic transport or dysfunction of interstitial protein processing [1]. Lymphedema can be primary or secondary, the former resulting from congenital abnormalities of the lymphatic system and the latter due to injury or dysfunction of lymphatic vessels or lymph nodes. Secondary lymphedema of the lower limb in cancer patients can develop after lymph node resection during surgery or lymphatic damage by radiotherapy [1].

Lymphedema can progress from mild to severe swelling with accompanying leakage of lymph from the skin and cellulitis; severe symptoms can adversely affect patients’ quality of life, ability to perform their activities of daily living, and psychosocial health [2–4]. Early and appropriate treatment is essential. Combined physical therapy (CPT) is the recommended treatment for lymphedema [1, 5, 6]. CPT consists of skin care, manual lymph drainage, compression therapy using multilayered bandages and elastic garments, and exercise. Although CPT is widely practiced in clinical settings, there is little evidence regarding its effects, especially for LLL [1, 5].

Active exercise with compression therapy (AECT) is recommended as part of CPT. Several reports have examined the effects of AECT on upper limb lymphedema (ULL). Concerning the long-term effects, Schmitz et al. [7] found that patients who lifted weights while wearing an elastic sleeve after breast cancer surgery showed improvements in lymphedema symptoms and muscle strength. Other studies have shown similar improvements in upper limb volumes after medium to long-term exercise interventions [8–10]. Exercise and compression have been shown to have immediate beneficial effects on edema. Concerning the immediate effects, Godoy et al. [11] compared edema before and after four 12-min sets of upper body exercises performed while wearing an elastic sleeve; they found a significant decrease in upper limb volume. Moseley et al. [12] reported that the edema-alleviating effect of AECT lasted 1 h when combined with deep breathing.

However, there are few reports of AECT for LLL. Katz et al. [13] reported that 5 months of weight lifting did not exacerbate lymphedema symptoms and improved muscular strength and walking ability, but no improvement in lower-limb volume was shown. The frequency, intensity, time, and type of AECT have not been determined properly. The only report of the immediate edema-relieving effects of exercise is our previous study [14], in which we demonstrated that 15 min of AECT in a seated position using a bicycle ergometer significantly decreased lower extremity volume. The ergometer allows muscle activity of the entire lower limb even at a relatively low load, but on the other hand, venous drainage while seated is less than when supine. Exercise in a supine position rather than in a seated position may result in greater lymphedema improvement because the synergistic effect of muscle pumping action during exercise and performing more venous return without gravity can be obtained. Thus, the purpose of this study was to determine the relative benefits of AECT with different postures for patients with LLL.

## Methods

### Study design

**Fig. 1:**
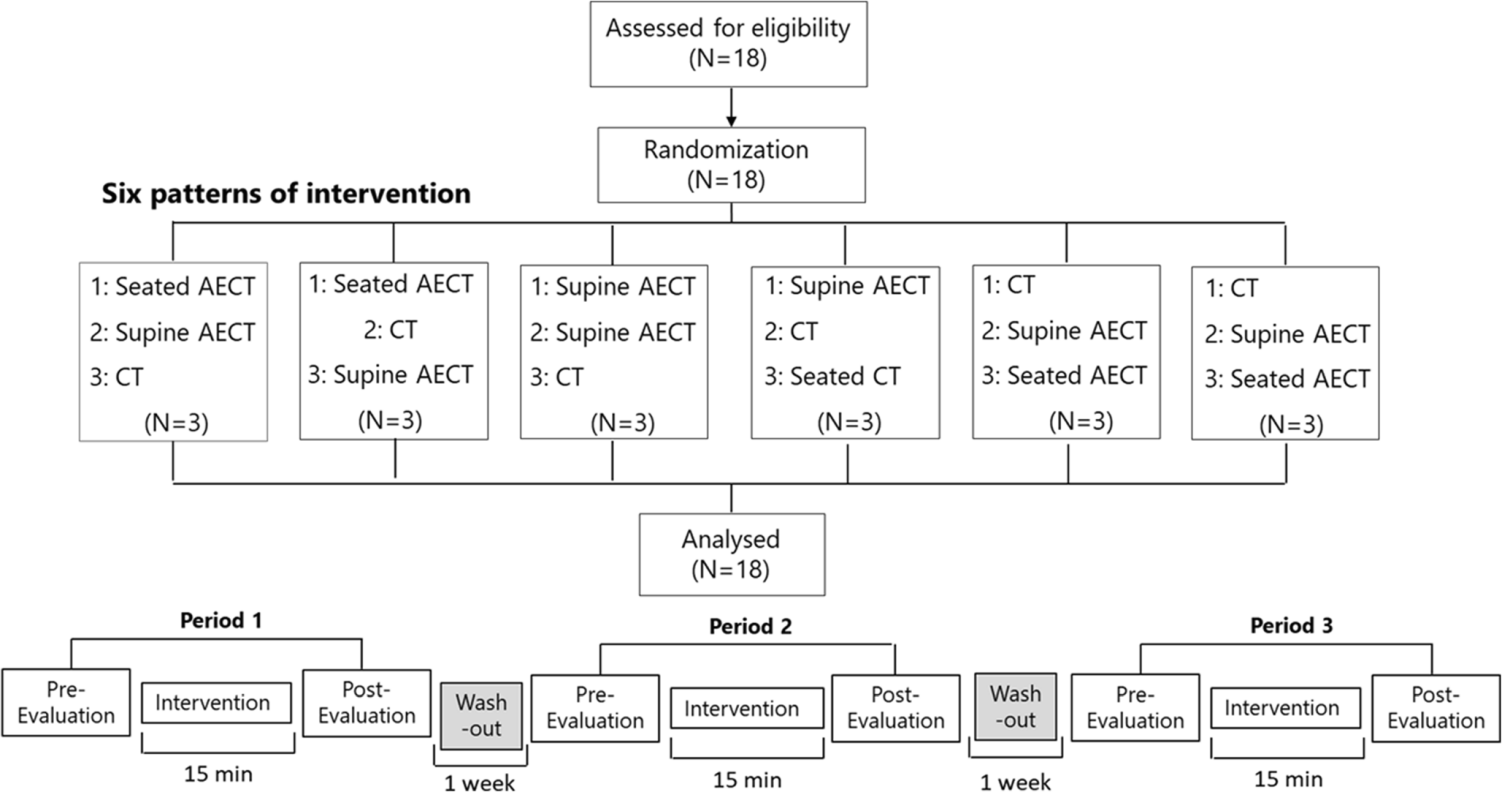
Study design: The trial had a randomized, controlled, crossover design. Each intervention was performed for 15 min, and the three interventions were separated by a ≥ 1-week washout period to eliminate any carryover effects. Measurements were taken before and after each intervention. Six patterns of intervention order were applied, with computer-generated randomization to eliminate order effects. *AECT, active exercise with compression therapy; seated AECT, AECT in a seated position; supine AECT, AECT in a supine position; CT, compression-only therapy

**Fig. 2:**
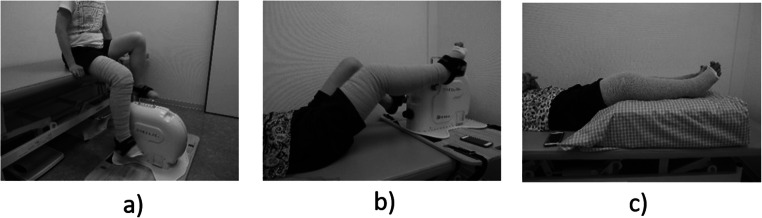
**a** Seated AECT is performed on a bicycle ergometer while wearing elastic compression bandages on the affected limb. **b** Supine AECT is performed on a bicycle ergometer while wearing elastic compression bandages on the affected limb. **c** T is performed with elevation of both legs while wearing elastic compression bandages on the affected limb. *AECT, active exercise with compression therapy; seated AECT, AECT in a seated position; supine AECT, AECT in a supine position; CT, compression-only therapy

Non-blinded intervention studies were conducted by block-randomized, crossover, comparison trials (3rd, 3rd intervention, 6 ways) (Fig. 1). The interventions were AECT in a seated position (seated AECT), AECT in a supine position (supine AECT), and compression-only therapy in a supine position (CT) (Fig. 2). AECT was performed on a bicycle ergometer, and CT was performed with elevation of both legs while wearing elastic compression bandages on the affected limb. Each intervention was performed for 15 min, and the three conditions were separated by a 1-week washout period. Participants performed normal self-care between the interventions.

Evaluations of primary and secondary endpoints were performed immediately before and after the intervention by trained examiners.

All participants were patient volunteers who were informed of the purpose, methods, and risks of the study and provided their written, informed consent before participating. This study was approved by the Keio University School of Medicine Ethics Committee (approval number: 2015-312) and conducted following the principles of the Declaration of Helsinki. The study was registered with the UMIN clinical trial registry (UMIN-CTR; ID000020129) by CONSORT 2010.

### Participants

Eligible patients (*N* = 18) with secondary lymphedema were recruited by rehabilitation physicians from cancer rehabilitation or lymphedema outpatient clinics at Keio University Hospital, Japan, from February 2016 to September 2009. The criteria of diagnosed gynecological cancer (stages I–IV), history of treatment for gynecologic cancer, stage II or greater LLL according to the International Society of Lymphology (ISL) lymphedema staging [1], 20–80 years of age, and ability to perform the exercise on a bicycle ergometer. The exclusion criteria of our study included patients had difficulty communicating, cellulitis, leakage of lymphatic fluid, deep venous thrombosis, severe aneurysm, severe heart disease, serious hepatic and renal dysfunction, or pulmonary embolism. Ethical approval for the study was received from the Keio University Ethics Committee. Each participant was informed about the study and provided written informed consent.

### Sample size

Based on past reports [17], the average lower-limb volume reduction after exercise was predicted to be 60 ml for the intervention group and 20 ml for the control group. To detect this difference with a two-sided significance level (*α* error) set to 0.05, a detection power of 80%, and the common standard deviation set to 60 ml, 17 patients were needed. The target number of cases was set to 18 patients to allow for trial dropouts.

### Outcome assessments

Participants were evaluated for lower extremity volume, general symptoms, and skin symptoms immediately before and after each intervention. Other clinical information (age, body mass index (BMI), cancer type, treatment, and ISL lymphedema stage [1]) was collected from medical records. ISL stages [1] were defined: stage I represents an early accumulation of fluid relatively high in protein content which subsides with limb elevation; stage II signifies that limb elevation alone rarely reduces the tissue swelling and pitting is manifest, later in stage II, the limb may not pit as excess subcutaneous fat and fibrosis develop; and stage III encompasses lymphostatic elephantiasis where pitting can be absent and trophic skin changes such as acanthosis, alterations in skin character and thickness, further deposition of fat and fibrosis, and warty overgrowths have developed.

### Primary endpoint: lower-limb volume

We measured the lower-limb volume using the Manual Perometer Type 1000M^TM^ (SN2110107, Pero-System, Germany) according to the protocol of Jeff et al [8]. The Perometer consists of a plate on which to place the foot to be measured, a movable frame surrounding it, a sensor inside the frame, a support to fix the frame and a movable handle, and uses infrared rays to create an image of the limbs. By putting the lower limb to be measured in the frame and standing, the lower limb blocks the infrared rays emitted from the inside of the frame, and the light receiving diode receives it, so that the outline of the limb can be drawn and the volume and circumference can be measured [15]. This has been reported as a very accurate method for assessing and measuring limb volume [16]. Both lower limbs were measured twice, and if there was a measurement error of 10% or more, the measurement was performed again. After the measurement, the data was taken into a personal computer (Lets note ^TM^, Panasonic, Windows Vista Business), and the lower-limb volume was calculated using Software PeroPlus^TM^ (manufactured by JUZO, Germany). The average value of the values measured twice before and after each intervention was adopted, and the percent change rate of the volume of each lower limb before and after the intervention was calculated.

### Secondary endpoint: general symptoms of pain and heaviness

The pain and heaviness of the lower limbs were measured using a visual analog scale (VAS). We used a method of marking the degree of pain on a horizontal straight line of 100 mm and quantifying the degree by the length [17, 18]. The left end is “no pain” and “no heaviness,” and the right end is “the highest pain you can imagine” and “the highest heaviness you can imagine”.

### Secondary endpoint: skin symptoms of skin stiffness and pitting edema

All assessments were performed by the same physical therapist who had received specialized lymphedema training. Skin hardness was evaluated by palpating the skin with the pad of the finger. The results were defined in 4 steps: 0: soft, 1: slightly hard, 2: moderately hard, and 3: extremely hard or fibrotic. Pitting edema was assessed by pressing the skin for 5 s. Zero was defined as no pitching, 1 was defined as pitching for 5 s or less, and 2 was defined as pitching for 5 s or more. Measurements were made at medial sites 10 cm proximal to the knee (AK10) and 10 cm distal to the knee (BK10), and on the dorsal aspect of the foot (FOOT).

### Interventions

The three interventions (Supine AECT, Seated AECT, and CT) performed by the participants in this study were conducted in the rehabilitation room and examination room of Keio Hospital (Fig. 2). In preparation for the three interventions, participants lay on their backs in bed and multilayered compression bandages were applied to the lower extremities. First, participants wore a stokinette (Tricofix®; BSN Medical, Luxembourg) on their feet, and then wrapped a padded foam bandage (Artiflex®; BSN Medical) around it. After that, a total of 6 short elastic bandages (Comprilan®; BSN Medical) of 8 cm × 2, 10 cm × 2, and 12 cm × 2 were wound up in order from the foot to the groin. All bandages were performed by the same physiotherapist. The calf sub-bandage pressure was confirmed using a Kikuhime ™ pressure sensor (TT Meditrade, Solo, Denmark) to maintain approximately 40 mmHg.

Concerning AECT, the intervention was performed using a variable-load ergometer (TE2- 20; Showa Denki Co., Ltd., Osaka, Japan) that allows pedaling while seated or supine. The ergometer was adjusted so that the knee was flexed to 30° and the hip to 60° in both the seated and supine positions. Before the exercise intervention, the exercise load was set by starting at the minimum load and increasing every minute until the target heart rate was reached; the load that achieved the target heart rate was used. If the target heart rate was not achieved, then the maximum load (20 W) was used. The target heart rate was determined using the Karvonen formula [19] with target heart rate = {(220 − age) − resting heart rate} × exercise intensity + resting heart rate. The exercise intensity was set to 0.3. Participants were given a 15-min break after load setting and then instructed to pedal the ergometer at 60 revolutions/minute for 15 min. A metronome was used to help participants maintain a constant rotation speed, and participants were verbally encouraged to pedal faster or slower as necessary.

Regarding CT, the participant lay supine with their legs elevated on an 18-cm-high leg-specific mat (MEDICS Co., Ltd., Tokushima, Japan) for 15 min.

All participants were instructed to maintain their usual lymphedema self-care management regimen, physical activity levels, and diet throughout the intervention period.

### Statistical analyses

All statistical analyses were performed on an intent-to-treat basis; data from all randomized patients were included in the analysis. Changes in lower extremity volume and general symptoms before and after the intervention were analyzed using the Wilcoxon signed rank test. ANOVA with linear mixed-effects modeling was used to compare and analyze the effects of the three interventions (seated AECT, supine AECT, and CT) and time (durations 1, 2, and 3). The effectiveness of the intervention was assessed using changes in least squares (LSM) percent of lower extremity volume and VAS scores for common symptoms (pain and severity). The McNemar-Bowker test was used to evaluate the effect of interventions on skin stiffness and dent edema. Spearman’s rank correlation was used to compare the severity of skin symptoms (rigidity and petechiae) before intervention with a decrease in lower extremity volume.

*P* values < 0.05 were considered significant. Data were analyzed using the Statistics version 23 software (IBM SPSS, Chicago, IL).

## Results

A total of 18 patients (18 limbs) were enrolled, and all completed the interventions and evaluations without any adverse effects. The mean patient age was 64.1 ± 10.8 years, and mean BMI was 23.2 ± 4.0 kg/m^2^. Lymphedema staging, performed according to the ISL criteria, was stage II in nine patients (50.0%) and late-stage II in nine patients (50.0%). The underlying pathology was endometrial cancer in five patients (27.8%), ovarian cancer in nine patients (50.0%), and cervical cancer in four patients (22.2%). All participants had surgery, 12 received chemotherapy, and two received radiation therapy.

### Lower-limb volume

Pre- to post-intervention changes are shown in Table 1. The LSM percentage changes in lower limb volume differed significantly among the three interventions (*P* = 0.011). Limb volume was significantly more reduced after supine AECT than after CT (*P* = 0.014), but limb volume changes after seated AECT and CT did not differ significantly (*P* = 1.000). There were no significant differences in limb volume between periods (*P* = 0.267), and the interaction between intervention and period was not significant (*P* = 0.147).

**Table 1:** Pre- to post-intervention changes in lower-limb volume and symptoms in patients with secondary lymphedema

Outcomes	Mean percentage change (95% CI)			*P*			
	Seated AECT	Supine AECT	CT	*P*1	*P*2	*P*3	Overall *P* value
Lower-limb volume	1.19% ± 1.32% (0.62–1.76%)	2.03% ± 1.24% (1.46–2.60%)	0.99% ± 0.83% (0.42–1.56%)	1.00	0.01	0.06	0.01
Pain	27.9% ± 53.2% (8.95–46.87%)	71.0% ± 22.4% (52.08–89.99%)	45.4% ± 28.8% (26.44–64.35%)	0.41	0.10	0.02	0.03
Heaviness	27.5% ± 63.6% (6.34–48.69%)	62.3% ± 26.2% (41.16–83.51%)	57.6% ± 27.6% (36.47–78.82%)	0.07	1.00	0.03	0.02

Note. Mean percentage changes in the least square mean (LSM) values were estimated on the basis of analyses of variance using linear mixed-effect modeling. LSM changes in lower limb volume and percentage changes in general symptoms of pain and heaviness were calculated as effects of the intervention.

*P1*, seated AECT versus CT; *P2*, supine AECT versus CT; *P3*, seated AECT versus supine AECT, *AECT*, active exercise with compression therapy; *seated AECT*, AECT in a seated position; *supine AECT*, AECT in a supine position; *CT*, compression-only therapy

### General symptoms of pain and heaviness

Pre- to post-intervention changes are shown in Table 1. The LSM percentage changes in pain differed significantly among the three interventions (*P* = 0.03). The improvement in pain was significantly greater with supine AECT than with seated AECT (*P* = 0.02). There were no significant differences between periods (*P* = 0.684), and the interaction between intervention and period was not significant (*P* = 0.707).

LSM percentage changes in heaviness differed significantly among the three interventions (*P* = 0.02). The improvement in heaviness was significantly greater with supine AECT than with seated AECT (*P* = 0.03). There were no significant differences between periods (*P* = 0.651), and the interaction between intervention and period was not significant (*P* = 0.370).

### Skin symptoms of skin stiffness and pitting edema

There were no significant changes in skin stiffness or pitting edema from before to after the three interventions at AK10, BK10, and FOOT.

### Correlation between severity of pre-intervention skin symptoms and lower-limb volume decrement

**Fig. 3:**
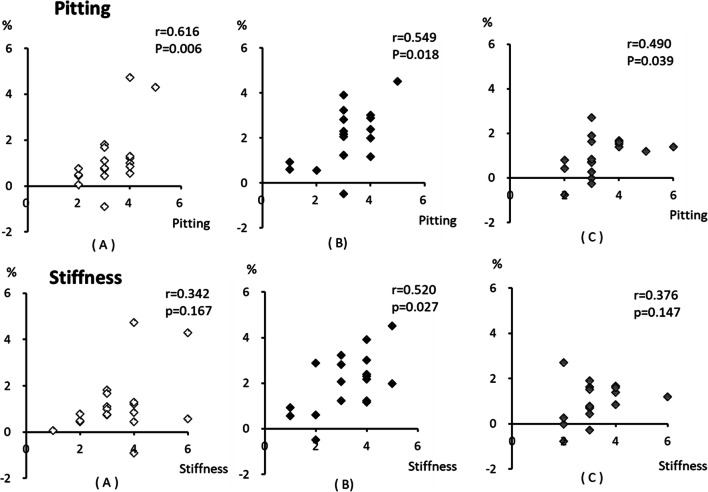
Correlations between severity of pre-intervention skin symptoms and percentage changes in lower-limb volume. The severity of pre-intervention skin pitting is significantly correlated with percentage changes in lower limb volume for seated (**a**) (*r* = 0.616, *P* = 0.006) and supine AECT (**b**) (*r* = 0.549, *P* = 0.018) and CT (**c**) (*r* = 0.490, *P* = 0.039). The severity of pre-intervention skin stiffness is significantly correlated with the percentage changes in lower-limb volume for supine AECT (**b**) (*r* = 0.520, *P* = 0.027), but not for seated AECT (**a**) (*r* = 0.342, P = 0.167) or CT (**b**) *(r* = 0.376, *P* = 0.147). *AECT, active exercise with compression therapy; seated AECT, AECT in a seated position; supine AECT, AECT in a supine position; CT, compression-only therapy

Figure 3 shows that the severity of pre-intervention skin stiffness correlated significantly with the percent changes in lower-limb volume for seated (*r* = 0.616, *P* = 0.006) and supine AECT (*r* = 0.5469, *P* = 0.018) and for CT (*r* = 0.490, *P* = 0.039), whereas the severity of pre-intervention pitting correlated significantly with percentage changes in lower-limb volume for supine AECT (*r* = 0.0520, *P* = 0.027), but not for seated AECT (*r* = 0.342, *P* = 0.167) and CT (*r* = 0.376, *P* = 0.147).

## Discussion

Our previous study [14] was the first report of a randomized, controlled, crossover trial to evaluate the immediate effects of AECT on LLL. We demonstrated the immediate edema-relieving effects in a seated position using a bicycle ergometer. Because venous drainage is less while seated than when supine, an exercise in a supine position might be more effective than in a seated position. Thus, the present study demonstrated the relative benefits of AECT with different postures for patients with LLL. Supine AECT using a bicycle ergometer was found to have marked immediate effects to decrease the fluid volume in severe LLL.

### Interventions

Multilayered compression bandages have been shown to have a greater positive effect on lymphatic drainage than elastic clothing [20]. The ISL recommends 60 mmHg as the maximum compression tolerable for lymphedema patients [1]. For stage II, late-stage II, and stage III lymphedema patients, a pressure of > 45 mmHg is recommended [5]. In this study, a compression pressure of 40 mmHg was used on the distal limb because a sub-bandage pressure exceeding 40 mmHg is recommended to counteract intravenous pressure in the antigravity position [21], and sub-bandage pressure rises during exercise [22]. In order to obtain consistent compression, the bandages were applied by the same person in all trials, and a pressure measurement device (KIKUHIME^TM^) that has been shown to have high reliability and validity [21] was used.

With regard to the exercise modality, the active exercise was then performed using a variable-load ergometer because it allows muscle activity of the whole lower limb even at low loads [23] and can be pedaled while seated or supine. The target heart rate was calculated using the Karvonen formula [19], and the exercise load amount was adjusted below the anaerobic threshold (AT). The AT is one of the indices of exercise intensity, and energy supply is provided by aerobic mechanisms at less than AT. Supine AECT is reported to generate an early anaerobic response because of the positional reduction in blood flow to the limbs. Therefore, an exercise intensity of 30% was selected so that all participants would be able to perform the exercise.

Previous studies have shown beneficial effects on limb volume of exercise sessions ranging from 15 to 60 min in duration [8, 10, 24, 25]. Goboy et al. [10] showed an immediate effect on ULL after a total of 48 min of exercise (four sets of 12 min each). In our previous study [11], an immediate effect on LLL was obtained after 15 min of exercise. Shorter exercise times are most likely to have good patient compliance in home-based therapy programs, so an exercise duration of 15 min was selected in the present study. The effects of exercise on ULL have been reported to last 1 h [12], but there are no data on the duration of exercise effects in LLL patients. For this reason, at least 1 week was allowed between exercise sessions to avoid potential cumulative effects.

### Lower-limb volume decrements and improvements in general symptoms

In the present study, as in our previous study [14], an immediate reduction in limb volume was observed after AECT. The present results show that supine AECT achieves greater limb volume reductions than seated AECT. Compression reduces edema by suppressing excessive extravasation of fluid, improving lymphatic vessel function, promoting lymph reabsorption, and enhancing the valve function of the lymphatic collecting ducts [26]. Exercising causes contraction and relaxation of the skeletal muscles and pulsatile compression of the veins and lymph vessels (muscle pump action), which stimulates lymphatic reflux and venous return and increases the carrying capacity of the lymphatics [5, 7–12]. The friction between the layers in multilayer bandages during exercise is thought to increase muscle pump action and promote effective drainage [27]. Even though a relatively low exercise load was applied in the present study, a significant volume reduction effect was obtained after both seated and supine AECT. The pedaling movements of the lower limbs combined with the compression of bandaging enhanced lymph flow and venous return. However, the reduction in limb volume was greatest after supine AECT. During seated AECT, the lower limb is below the heart, resulting in a venous pressure gradient that can exacerbate edema. Lymph flow out of the lower limbs is enhanced by gravity when supine, and venous return is increased. Elevation has been previously shown to partially mitigate lymphedema [28]. The present results support this and indicate that further benefits are obtained when the affected limbs are compression bandaged and exercised.

As in our previous LLL study [14], the present study also found immediate improvements in general symptoms of pain and heaviness after AECT. The causes of pain in lymphedema include inflammation, tissue stretching, ischemia, complex regional pain syndrome (CRPS), and recurrence/progression of cancer [5]. Since fluid accumulation likely increases the weight of the lower limb, it is possible that symptom amelioration may be partially due to a decrease in the weight of the affected limb. Reductions in feelings of tightness may also be due to decreased limb volume and to the stretching effects of the pedaling motion. There was a significantly greater reduction in feelings of heaviness and pain after supine than after seated AECT. This suggests that the symptom improvements are more likely due to reductions in fluid in the limb than to the stretching motions of the exercise.

### Correlations between severity of pre-intervention skin symptoms and lower-limb volume decrements

Skin hardening indicates fibrosis, which is associated with the progression of lymphedema. In the early stage of lymphedema, the subcutaneous tissues can be compressed so that a mark remains, but in the late stage of lymphedema, the fluid content of the tissue decreases, and fibrous and adipose tissue contents increase, resulting in hardened skin that cannot be pitted by compression. Early in this process, the skin keeps its flexibility; however, protein accumulation leads to fibrous and adipose tissue proliferation, and in ISL stage III lymphedema, these symptoms progress to elephantiasis [1].

Although there were no significant changes in skin hardness and indentation after any of the interventions, the degree of indentation before the intervention was significantly positively correlated with the amount of lower-limb volume reduction after all three interventions. In contrast, the degree of skin hardness before the intervention was significantly positively correlated with the reduction in lower-limb volume only after supine AECT. This suggests that patients with severe lymphedema and advanced skin hardness may benefit more from AECT while recumbent to reduce lower-limb volume. In late ISL stage II, when skin hardening has become severe, the shape of the lymph ducts changes [29], and their lumens narrow. Once this occurs, the pumping action of muscle during exercise becomes less effective at improving lymphatic drainage. Therefore, using gravity to enhance the effects of exercise and compression by having the patient assume a supine position achieves a better result than exercise and compression while the patient is upright. The results of the present study are clinically important for designing treatment programs.

### Implications for future research and limitations

A limitation of this study is that the scale used to evaluate skin hardness and indentation was a subjective clinical scale based on palpation. Quantitative evaluation of these parameters would be helpful, and we hope that, in the future, instruments capable of quantitatively measuring these factors will be developed. In addition, this study examined the immediate effects of exercise therapy on LLL, but future work should also investigate long-term intervention effects. Finally, other exercise modes, durations, frequencies, and intensities should be tested to determine the optimal treatment for LLL.

## Data Availability

The datasets during and/or analyzed during the current study are available from the corresponding author on reasonable request.
